# Adjuvant Systemic Therapy in Older Breast Cancer Women: Can We Optimize the Level of Care?

**DOI:** 10.3390/cancers7030833

**Published:** 2015-07-03

**Authors:** Anna Rachelle Mislang, Laura Biganzoli

**Affiliations:** Sandro Pitigliani Medical Oncology Unit, Nuovo Ospedale Santo Stefano, Instituto Toscano Tumori, 59100 Prato, Italy

**Keywords:** breast cancer, elderly, fitness for treatment, decision-making, geriatric oncology, adjuvant therapy, geriatric assessment

## Abstract

Defining optimal adjuvant treatment for older women with breast cancer is challenged by the lack of level-1 clinical evidence and the heterogeneity of the older population. Nevertheless, recommendations based on reviews of available evidence mainly from retrospective subgroup analyses and extrapolation of study results from younger patients, and expert opinions, may be useful to guide treatment decisions in fit patients. But how can we properly define a “fit” older patient? In clinical practice, age by itself and clinical impression generally drive treatment decision, although the appropriateness of this judgment is under-documented. Such an approach risks overtreatment or, more frequently, undertreatment. A geriatric assessment can be valuable in oncology practice to address this issue. In this review article, we will focus only on systemic treatment and will discuss “standard” adjuvant systemic treatment strategies for fit older breast cancer patients and the role of “personalized” systemic therapy in unfit patients. The concepts conveyed in this review cannot be extrapolated to locoregional therapy.

## 1. Introduction

The median age for breast cancer diagnosis is around 60 years and over 40% of all breast cancers are diagnosed in women aged 65 years or older [1]. Due to the aging population, it is expected that the proportion of older women with early breast cancer will grow considerably in the near future [2]. There is sufficient evidence suggesting that older cancer patients do just as well as younger patients when offered standard adjuvant treatment. Despite this, there remains minimal consensus on how this group should be treated, primarily from insufficient evidence either due to lack of representation or poor accrual in clinical trials. This may frequently lead to under-treatment or, less commonly, overtreatment of the patient with subsequent poor outcomes.

## 2. Breast Cancer in the Elderly

Breast cancer death rates in both United States and European populations have declined during the past decade. However, there is a preferential improvement observed in younger women of less than 75 years [3,4] despite the generally favorable tumor biology in the older population [5]. This is most likely related to underuse of standard adjuvant treatments (either from lack of clinical evidence, physician-bias, or patient-bias) linked to higher mortality rates in older cancer patients. Observational studies have associated under-treatment with worse breast cancer outcomes [6,7] and age is an independent risk factor for receiving less than standard treatment [6]. The treatment approach in the elderly is never straightforward and requires careful consideration of age heterogeneity, competing comorbidities, and estimated life expectancy. Comorbidities increase with age and are correlated with significant decrease in life expectancy irrespective of specific cancer diagnosis [7]. Little benefit is expected from adjuvant treatment in women with multiple competing illnesses. Whereas for healthy older women, breast cancer poses a significant threat to their lives and, as such, standard adjuvant treatment should ideally be recommended.

### Identifying Who Is “Fit” for Treatment: The Role of Comprehensive Geriatric Assessment (CGA)

The benefit of giving cancer therapy to the elderly who are likely to die prematurely from non-cancer-related causes is questionable; however, it is challenging for clinicians to easily identify these patients. Given the heterogeneity of the elderly population, offering treatment based on chronological age alone is never substantiated. Instead, biological age—which refers to the presence of comorbidities and the general fitness or health—should be used. Combined geriatric and oncology management may improve estimation of life expectancy and prediction of treatment tolerance [8,9,10]. CGA is a tool performed through collaboration with the multi-disciplinary geriatric team that aims to provide a systematic, evidenced-based and reproducible health assessment, and to subsequently guide focused geriatric interventions and appropriate oncologic treatment decisions [11,12,13,14,15]. Evidence shows that the implementation of CGA to identify and guide management of reversible domains in geriatric patients (particularly comorbidities, depression and nutrition), subsequently improves compliance, treatment tolerability, quality of life (QoL) and survival [10]. In geriatric oncology, CGA has been shown to add further information on patient fitness over that of performance status [16], be an independent predictor of survival irrespective of tumor type or performance status [17], predict outcomes with curative chemotherapy when utilized to stratify level of fitness of patients (fit *vs.* unfit was better than clinical assessment) [18], and identify older breast cancer patients who are potentially fit enough for adjuvant chemotherapy, but otherwise would not be treated based on chronological age [19]. Balducci has previously proposed an algorithm for the management of older cancer patients based on their level of fitness on CGA [15]. He defined “fit” patients as those who lacked severe comorbidities and were functionally independent while “frail” patients as those who were functionally impaired, and/or had severe comorbidities and/or geriatric syndromes. Patients with intermediate characteristics were defined as vulnerable. Recently, the International Society of Geriatric Oncology (SIOG) composed a panel with expertise in geriatric oncology to develop consensus statements on the use of CGA in older patients with cancer [14]. The panel recommended evaluation of the following domains in the geriatric assessment: functional status, comorbidity, cognition, mental health status, fatigue, social status and support, nutrition, and presence of geriatric syndromes. The panel concluded that CGA could be valuable in oncology practice for detection of impairment not identified in routine history or physical examination, estimation of survival in a variety of tumors and treatment settings, and prediction of severe treatment-related toxicity to influence treatment choice and intensity [14]. Identifying patients that would benefit from CGA is still an area of controversy. Performing CGA is time-consuming and therefore not always practical to perform in all elderly patients. Hence the use of screening tools, although not designed to replace CGA, may be used to provide a quicker, alternative means to identify unfit patients in need of a full CGA and guided multidisciplinary intervention [20].

**Table 1:** Predictors of chemotherapy toxicity in the elderly.

CRASH	CARG
Hematologic Scores ○ Diastolic BP ○ IADL ○ LDH ○ Chemotox Non-hematologic Scores ○ ECOG PS ○ MMS ○ MNA ○ Chemotox	Age >72 years Cancer type: GI or GU Standard chemotherapy dosing Polychemotherapy (>1 chemotherapy drug) Hemoglobin ○ <10 g/dL (females) ○ <11 g/dL (males) Creatinine clearance <34 mL/min (Jelliffe) Hearing impairment, fair or worse Functional impairment ○ >1 fall in 6 months ○ IADL: some help or unable to take medications ○ MOS: limited walking to 1 block ○ MOS: decreased social activities due to physical or emotional health

BP: Blood pressure; IADL: Instrumental activities of daily living; LDH: Lactate dehydrogenase; ULN: Upper limit of normal; Chemotox: Chemotoxicity of the regimens were calculated using an index to estimate the average per-patient risk of chemotherapy toxicity; ECOG PS: Eastern Cooperative Oncology Group performance status; MMS: Mini Mental Status; MNA: Mini Nutritional assessment; GI: Gastrointestinal; GU: Genitourinary; MOS: Medical outcomes study.

Two prospective, multicentre studies developed a scoring system to predict the risk of chemotherapy toxicity in elderly patients [21,22]. The Chemotherapy Risk Assessment Scale for High-age patients (CRASH) score utilized the MAX2 index to predict the average risk for developing severe hematological and non-hematological toxicities from various chemotherapy regimens (chemotox) in patients aged >70 years [23]. Similarly, the Cancer and Aging Research Group (CARG) developed a risk stratification scheme and found 11 risk factors associated with increased risk for chemotherapy toxicity in older adults with cancer [22]. Both studies provided an objective risk estimate that may be useful in assisting with decision-making or may be even used for stratifying patients in the clinical trials. The predictive risk factors identified from these studies are listed in Table 1.

## 3. Adjuvant Chemotherapy

There is mounting evidence that fit, older cancer patients do benefit from adjuvant chemotherapy. In older women with estrogen receptor (ER)-negative tumors, the benefit from chemotherapy is likely to be as high as that of younger patients [24,25,26]. However, the same benefit is less evident with ER-positive tumors. In a review of data from the National Cancer Institute Surveillance Epidemiology and End Results (SEER) database from 1991 to 1999 that included over 40,000 women aged 65 years or older with diagnosis of early stage breast cancer, 4500 (11%) were recorded as receiving chemotherapy. Women with node positive, ER-negative disease, gained a statistically significant benefit from chemotherapy for both breast cancer specific survival (BCSS) (hazard ratio (HR) = 0.72, 95% confidence interval (CI) 0.54–0.96) and overall survival (OS) (HR = 0.65, 95% CI 0.52–0.82) while no evident benefit was seen in ER-positive breast cancers [26]. Similarly, in an observational study of 1711 women aged ≥66 years with ER-negative breast cancer, multivariate regression analysis showed that chemotherapy conferred a 15% reduction in risk of death from any cause, compared with patients not treated with chemotherapy (HR = 0.83, 95% CI 0.74–0.92) [25].

A few adjuvant studies have been conducted specifically in older breast cancer patients, where the majority presented with high-risk tumors (Table 2).

**Table 2:** Adjuvant chemotherapy clinical trials focused on older breast cancer patients.

Trial	Age Cut-Off (Median)	Sample Size, *n*	Treatment Arms	Tumour Characteristics	Outcomes
**FASG 08**	>65 (69)	338	Tam +/− Epi	Node +	No ≠ in DFS; ↑ DFS with Epi + Tam at multivariate analysis
**CALGB 49907**	>65	633	CMF or AC *vs.* capecitabine	Stage I-IIIB	↑ DFS and OS with AC/CMF
**ELDA**	>65 (71)	299	CMF *vs.* weekly docetaxel	Node + or average to high-risk ^1^ for recurrence (Hormone Receptor −, G 2–3, or T > 2 cm)	Docetaxel no more effective than CMF; worse QoL and more side effects
**ICE**	>65 (71)	1358	Ibandronate +/− capecitabine	Node + or high-risk ^2^ node −	No ≠ in DFS or OS
**ICE II**	>65	207	EC or CMF *vs.* PX	Increased risk ^3^	No ≠ in DFS; EC/CMF better tolerated
**CASA**	>65	77	Nil *vs.* PLD *vs.* metronomic CM	Hormone Receptor −	Worse QoL, deterioration of cognitive and physical functioning with PLD

Tam: Tamoxifen; Epi: Epirubicin; DFS: Disease free survival; OS: Over-all survival; CMF: Cyclophosphamide, methotrexate and fluorouracil; AC; doxorubicin and cyclophosphamide; QoL: Quality of life; EC: Epirubicin + cyclophosphamide; PX: Nab-Paclitaxel + capecitabine; PLD: Pegylated liposomal doxorubicin; CM: Cyclophosphamide + methotrexate; ORR: Over-all response rate; ≠: Difference; >: Greater than; ↑: Improved; G: Grade; T: Tumor size; +/−: With or without; +: Positive; −: Negative. ^1^ Estrogen and progesterone receptor negative or histologic grading 2–3 or primary tumor >2 cm; ^2^ Node negative disease with at least 1 other risk factor (tumor size >2 cm, grade 2 or 3, ER and PR negative); ^3^ As determined by urokinase-type plasminogen activator/plasminogen activator-1 or clinic-pathological risk parameters.

### 3.1. Clinical Trials Evaluating the Role of Chemotherapy in Older Women with Breast Cancers

In the French Adjuvant Study Group 08 (FASG 08) trial, fit elderly women aged 65 years and above with node positive early breast cancer, were randomized to tamoxifen with or without weekly epirubicin [27]. The six-year disease free survival (DFS) showed a non-statistically significant improvement (72.6% *vs.* 69.3% *p* = 0.14) in favor of combination arm; the relative risk of relapse in multivariate analysis was significantly higher in patients who received tamoxifen alone, compared with patients treated in combination (HR 1.93, *p* = 0.005) [27].

The Cancer and Leukemia Group B (CALGB) 49907 trial compared the use of standard polychemotherapy with doxorubicin/cyclophosphamide (AC) or cyclophosphamide/methotrexate/fluorouracil (CMF) with single agent capecitabine as adjuvant treatment for elderly women, aged ≥65 years with stage I to IIIB breast cancer. This trial was designed to demonstrate the non-inferiority of a “more gentle” chemotherapy option in older women. The first interim analysis on 600 enrolled patients showed a twofold risk of recurrence or death with capecitabine (HR 2.09, *p* ≤ 0.001) and a marked improvement of three-year DFS (85% *vs.* 68%, *p* ≤ 0.001) and OS (91% *vs.* 86%, *p* = 0.02) rates with the use of polychemotherapy over capecitabine, confirming its superiority over the latter [28]. In a subgroup analysis accounting for ER status, the superiority in DFS and OS of standard chemotherapy was evident only in ER negative patients but no definite conclusions can be drawn, as this was unplanned post hoc analysis. Of note, only 4% of enrolled patients were 80 years or older, thereby also limiting conclusions in this particular age group. Nevertheless, this trial not only showed that adjuvant chemotherapy improves survival in older women but also provided the first prospective evidence that carefully selected fit older patients derive as much benefit from standard adjuvant chemotherapy as younger patients, and that attenuation of treatment to prevent side effects can lead to poor survival outcomes.

Results from the pre-planned QoL sub-study of CALGB 49907 trial confirmed that monotherapy capecitabine had far less toxicity than the standard polychemotherapy (CMF or AC), in terms of nausea, vomiting, constipation, fatigue and psychological stress—leading to a better global QoL scores [29]. However, this difference was only transient and was completely resolved after 12 months from baseline, arguing that the short-term toxicity from the standard polychemotherapy may be an acceptable trade-off for an enhanced survival advantage.

Elderly Docetaxel Adjuvant (ELDA) randomized elderly women aged ≥65 years, with node positive or deemed average-to-high-risk node negative tumors (*i.e.*, ER and PR (progesterone receptor) negative or histologic grading 2–3 or primary tumor >2 cm), to either standard CMF or weekly docetaxel [30]. No statistically significant difference in terms of DFS and OS was observed between the two treatment arms; unadjusted HR of DFS and OS for docetaxel *vs.* CMF were 1.21 (95% CI 0.83–1.76, *p* = 0.32) and 1.34 (95% CI 0.80–2.22, *p* = 0.26) respectively [30]. However, the pattern of toxicity was significantly different. Compared with standard CMF, weekly docetaxel caused less nausea, mucositis, and hematological toxicity, but more allergy, fatigue, hair loss, onychopathy, dysgeusia, diarrhea, abdominal pain, neuropathy, cardiac and skin toxicity [30].

Three toxic deaths were reported: one with CMF and two with docetaxel. Notably, QoL was worse with docetaxel in many parameters including change in body image, decline in future perspective, and aggravated non-hematological side effects. Increasing age, functional impairment, number of comorbidities and docetaxel treatment were independently associated with severe non-hematological toxicity in this population of 65–79 year olds. The authors therefore concluded that weekly docetaxel could not be considered a standard adjuvant regimen in older breast cancer patients based on its adverse safety profile, negative impact on QoL and absence of superior efficacy over standard CMF.

The Ibandronate with or without Capecitabine in Elderly Patients with Early Breast Cancer—(ICE Study), prospectively studied 1358 patients aged ≥65 years, with node-positive or high-risk (at least one of the following: tumor size ≥2 cm, Grade 2–3, ER and PR negative) node-negative early-stage breast cancer who were deemed inappropriate for conventional treatment. Patients were randomized 1:1 to receive capecitabine plus ibandronate or ibandronate alone for two years. Results showed no difference between the two treatment arms for the primary endpoint of three-year invasive DFS: 85.4% in women treated with capecitabine plus ibandronate *vs.* 84.3% in the ibandronate-alone arm; the five-year invasive DFS was 78.8% *vs.* 75%, respectively. Similarly, there was no difference in OS at the end of three years (95% *vs.* 94%) and five years (90% *vs.* 88%) [31]. The negative results of ICE study did not favor the use of capecitabine monotherapy, thus complementing the results of CALGB 49907 in supporting the use of combination chemotherapy as a standard adjuvant treatment for elderly women over that of capecitabine alone.

The ICE II trial randomized 400 non frail women, aged ≥65 years with high risk features (as determined by urokinase-type plasminogen activator/plasminogen activator inhibitor-1 or clinic-pathological risk parameters) breast cancer to standard arm—four cycles of EC or six cycles of CMF *vs.* experimental arm—six cycles of weekly nab-paclitaxel plus capecitabine (PX) [32]. Interim safety analysis performed after 207 patients completed the treatment revealed that EC or CMF was more tolerable than PX. Compared to the standard arm, more patients in the experimental arm left the study due to adverse events (6.6% *vs.* 34.7%). Although standard arm had more non-hematological adverse events than experimental arm (58.8% *vs.* 18.7%, *p* = 0.001), grade 3–4 non-hematological adverse events were higher in PX group. The rates of invasive DFS were equivalent between the two arms (HR 0.98, *p* = 0.9597) at 48 months [32].

Data on the role of adjuvant chemotherapy in older breast cancer patients were also extrapolated by subgroup analyses from studies conducted in the general population. The U.S. Oncology 9735 study compared adjuvant AC with docetaxel/cyclophosphamide (TC). After seven years median follow up, patient cohorts treated with TC resulted in a significant benefit over AC for both DFS (HR = 0.74, *p* = 0.033) and OS (HR = 0.69, *p* = 0.032) [33,34]. In this trial, 16% of enrolled patients were aged 65 years or older and subgroup analysis showed that the benefit from TC was maintained in the older age group [34].

### 3.2. Patterns of Toxicity of Adjuvant Chemotherapy in Older vs. Younger Patients

Although it is clear that adjuvant chemotherapy has improved outcomes in the elderly with high risk early breast cancer and that healthy older patients could theoretically receive similar regimens offered to younger patients, elderly patients should be carefully monitored as they are more vulnerable to treatment toxicities [35]. Compelling data from two large population-based studies highlighted the increased risk of anthracycline-related toxicities that are specifically relevant to older cancer patients due to concerns of congestive heart failure (CHF) and myelodysplastic syndrome (MDS) or acute myeloid leukemia (AML) [36,37]. In the US Oncology 9735 study, hematological toxicities were doubled within the older age subgroup of ≥65 years: More febrile neutropenia with TC (8% *vs.* 4%) and more anemia with AC (5% *vs.* 2.5%) [34], thereby further emphasizing the need for careful monitoring in these patients. All 3 late deaths (that were likely chemotherapy-related) were noted in the AC arm, favoring use of non-anthracycline-based regimen in the elderly patients, if possible.

Several adjuvant studies have also demonstrated increased toxicity risk in the older subgroup [28,29,38,39,40]. The International Breast Cancer Study Group (IBCSG) VII trial compared tamoxifen with or without three cycles of CMF in 299 node-positive breast cancer patients, of whom 76 (25%) were aged 65 years or above. A higher incidence of grade 3 toxicities of any type were observed in the ≥65 group *vs.* <65 group (17 *vs.* 7%) though there was no difference seen in the QoL as measured by performance status, coping, physical well being, mood, and appetite between the 2-subgroups [39]. Joint analyses of IBCSG trials I-IX showed an increased incidence of CMF-related deaths in patients aged 65 years or older, and that deaths related to sepsis or toxicity were directly correlated with aging [40].

An analysis of three large adjuvant clinical trials from CALGB compared the toxicity and over-all tolerability of anthracycline-based treatment in older and younger patients [38]. These three trials were: CALGB 8541—a comparison of cyclophosphamide, doxorubicin, and fluorouracil (FAC) in three dose schedules; CALGB 9344-AC with or without paclitaxel; and CALGB 9741-AC and paclitaxel as standard *vs.* dense dosing. Toxicity data were evaluable in 93% of 6642 enrolled patients, where 7% were age ≥65 and 3% were age ≥70 years. On multivariate analysis, older patients had a higher rate of hematological toxicity and treatment related deaths than younger patients, with no differential non-hematological toxicity. In addition, elderly groups were significantly more likely to have treatment cessations due to toxicity, or to die of AML/MDS [38].

Based on the efficacy and safety data discussed, it is recommended to offer adjuvant chemotherapy for fit older breast cancer patients with node-positive (N+) ER-negative disease and to consider chemotherapy for patients with node-negative (N0) ER-negative disease, as well as for patients with ER-positive disease, if additional unfavorable features (high tumor burden *i.e.*, ≥4 positive nodes, high risk on genetic-based assay or classical biological features) are present [41]. Polychemotherapy remains the standard optimal treatment offering superior survival outcomes and is therefore preferable over single agent chemotherapy [28]. However, higher rates of toxicity could tip the balance away from using combination chemotherapy depending on the patient’s fitness level and disease characteristics. The patterns of toxicity observed in older breast cancer patients treated with polychemotherapy regimens in comparison with those observed younger patients are listed in Table 3.

**Table 3:** Toxicity risks of adjuvant polychemotherapy in older cancer patients.

Trial Name	Regime	Age Years: % Population	Toxicity in the Elderly
US Oncology 9735	TC *vs.* AC	≥65: 16%	TC: 2-fold risk of febrile neutropenia AC: 2-fold risk of anemia
IBCSG	Tam +/− CMF	≥65: 25%	CMF higher risk for Gr 3 toxicity of any type; mostly hematological and mucosal
CALGB 8541 CALGB 9344 CALGB 9741	FAC AC +/− paclitaxel AC + P standard *vs.* dense dose	≥65: 7% ≥70: 3%	Higher hematological toxicities, more treatment cessations

TC: Docetaxel and cyclophosphamide; AC; doxorubicin and cyclophosphamide; Tam: Tamoxifen; CMF: Cyclophosphamide, methotrexate and fluorouracil; +/−: With or without; >: Equal or greater than; Gr: Grade.

The benefit of chemotherapy has only been investigated in fit patients leaving its role in vulnerable patients ambiguous. The Chemotherapy Adjuvant Study for women with advanced Age (CASA) trial studied elderly women aged ≥65 years with endocrine non-responsive (ER and PR negative) breast cancer who were not suitable to receive standard chemotherapy regimen [42]. Patients were randomized to receive no treatment (CASA nil) or active treatment consisting of liposomal doxorubicin (PLD) or low dose, metronomic cyclophosphamide plus methotrexate (CM) for 16 weeks. Overall, patients on PLD reported worse QoL, cognitive and functional decline than non-PLD regimens (primarily CM). Among the 72 patients who started treatment, 70 (97%) patients developed some type of adverse event, where 19 (51%) on PLD group and 12 (34%) on CM group experienced grade 3 adverse events. The most common grade 3 adverse events were hand-foot skin reaction on PLD (eight cases) and hypertension on CM (7 cases); there were no grade 4 or 5 adverse events reported during treatment on both arms. At a median follow-up of 42 months, 78% of randomized patients remained free of any breast cancer recurrence [42]. The trial however, is limited by inadequate accrual (only 77 out of estimated 1296 or 6% of the target sample) precluding any conclusion on the role of PLD or CM as adjuvant treatment for vulnerable older breast cancer patients.

Although not specifically conducted in older or unfit patients, the result of the CALGB 40101 trial may be relevant to these populations. The trial addressed the non-inferiority of single-agent paclitaxel (T) to AC, both given for 4 or 6 cycles to 3871 patients with early breast cancer. The study design was based on the premise that an optimal adjuvant chemotherapy for early-stage breast cancer balances efficacy and toxicity [43]. The study was unable to conclude non-inferiority of T with AC. At a median follow-up period of 6.1 years, the results showed HR 1.26 for relapse free survival (RFS) and HR 1.27 for OS—both favoring use of AC over T. The estimated absolute advantage of AC over T at 5 years was 3% for RFS (91% *vs.* 88%) and 1% for OS (95% *vs.* 94%). As expected, the incidence of any grade 3 or higher hematologic toxicity was considerably higher in the AC arm compared with the T arm; all nine treatment-related deaths (two cardiac deaths and seven secondary hematological malignancies) were observed in patients treated with AC. Based on these results, weekly paclitaxel cannot be considered a standard option in patients who are fit enough to receive the standard polychemotherapy. However, the limited absolute difference in survival between the two treatment arms and the better safety profile of T *vs.* the standard regimen (AC) make single agent paclitaxel a reasonable option at least for high risk patients, who are considered not eligible for standard chemotherapy.

**Figure 1:**
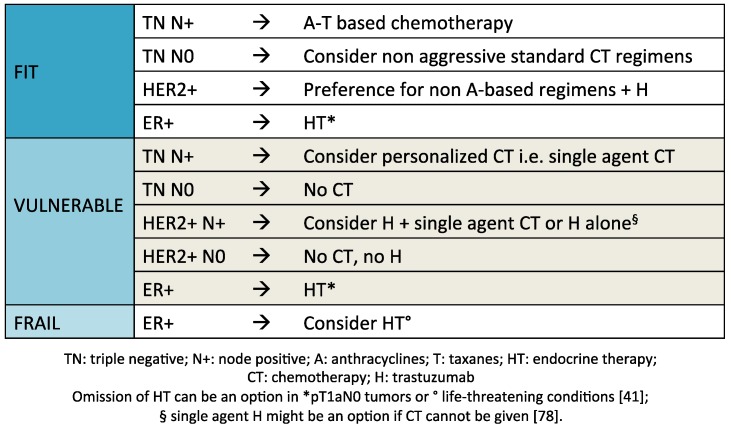
Proposed algorithm for adjuvant systemic therapy in older breast cancer patients based on the level of fitness and tumor biology.

Treatment algorithm for adjuvant systemic therapy in older breast cancer patients is shown in Figure 1.

### 3.3. Role of Primary Prophylaxis with Granulocyte Colony Stimulating Factors (G-CSF) in Elderly Women with Breast Cancers on Chemotherapy

Chemotherapy-induced neutropenia is a major risk factor for infection-related morbidity and mortality as well as for a significant dose-limiting toxicity in cancer treatment. Patients developing severe (grade 3/4) febrile neutropenia (FN) during chemotherapy often leads to treatment reductions, delays or omissions. This may impact the success of treatment, particularly when treatment intent is either curative or to prolong survival. Since the risk of myelosuppression is increased with aging, an adequate use of G-CSF is particularly relevant in the older population. Updated guidelines from the National Comprehensive Cancer Network (NCCN) and the European Organisation for Research and Treatment of Cancer (EORTC) both recommend the use of primary prophylaxis with G-CSF to prevent FN in patients who are high (>20%) or intermediate (10%–20%) risk, based on age (>65) and presence of other patient-related adverse risk factors (*i.e.*, extensive prior chemotherapy, poor performance status, pre-existing neutropenia, poor nutritional status, previous irradiation and advanced disease) [44,45,46].

## 4. Adjuvant Trastuzumab

Adjuvant trials have demonstrated significant reductions in disease recurrence and improvement in survival for patients with early-stage, Her-2 positive breast cancers treated with trastuzumab-based chemotherapy [47,48,49,50,51,52]. One year of trastuzumab, preferably given in combination rather than in sequence with chemotherapy, represents the standard of care [53,54]. It is worthwhile noting though, that there are almost no older patients included in these adjuvant trastuzumab trials. Less than 20% of the patients included in the HERA and NSABP B31/N9831 trials were aged 60 or above, and the benefits of trastuzumab in this subgroup were presumed to be similar as the whole study population [49,55,56]. Interestingly, in a retrospective analysis of HERA, age was not strongly associated with prediction of benefit from trastuzumab therapy [57].

Both the SIOG and the European Society of Breast Cancer Specialists (EUSOMA) recommend that all patients with HER2-positive breast cancer and without cardiac disease should be offered trastuzumab in combination with chemotherapy [41].

### 4.1. The Risk of Trastuzumab-Related Cardiotoxicity in the Elderly

Clearly, trastuzumab use is associated with an increased risk of cardiotoxicity. The overall incidence varies according to the definition used, the cytotoxic partners (anthracycline *vs.* no anthracycline) and sequence of treatment (whether given concomitantly or sequentially with chemotherapy) [58]. In several pivotal adjuvant clinical trials, the rates of symptomatic CHF ranged from 1.5% to 5.1%, and the rates of decreased left ventricular ejection fraction (LVEF) ranged from to 3.5% to 19% [47,48,49,59,60,61,62,63,64].

In the independent adjuvant cardiac review of symptomatic heart failure events in the NSABP B31/N9831, age older than 50 years was an independent predictor for cardiac events [65]. Whereas in the HERA trial, no difference in the incidence of cardiac events between age <60 *vs.* ≥60 years subgroup was found [66].

In a systematic review of prospective randomized trials (with available data on the use of adjuvant trastuzumab in patients older than 60 years) by Brollo *et al.*, a significant 47% relative risk reduction and 5% cardiac events were observed in elderly patients receiving trastuzumab compared to chemotherapy alone [67]. Using SEER-Medicare data from 2000 through 2007, Chen *et al.* identified 45,537 women aged 67 to 94 years with early-stage breast cancer and calculated three-year incidence rates of CHF or cardiomyopathy (CMP) for the following treatment groups: Trastuzumab (with or without non-anthracycline chemotherapy), anthracycline plus trastuzumab, anthracycline (without trastuzumab and with or without non-anthracycline chemotherapy), other non-anthracycline chemotherapy, or no adjuvant chemotherapy or trastuzumab therapy. Adjusted three-year CHF or CMP incidence rates were higher for patients receiving trastuzumab (32.1 per 100 patients) and anthracycline plus trastuzumab (41.9 per 100 patients) compared with no adjuvant therapy (18.1 per 100 patients, *p* < 0.001) [68].

Chavez-MacGregor *et al.* looked at trastuzumab-related cardiotoxicity among patients aged ≥66 years diagnosed with stage I-III breast cancer between 2005 and 2009 and treated with chemotherapy. The median age of the cohort was 71 years and out of 9535 patients identified in SEER-Medicare and in the Texas Cancer Registry-Medicare databases, 2203 (23.1%) received trastuzumab. The rate of CHF was higher in trastuzumab *vs.* non-trastuzumab users (29.4% *vs.* 18.9%, *p* < 0.001); among trastuzumab-treated patients, the following factors increased the risk of CHF: Older age (age > 80 years; HR 1.53; 95% CI 1.16–2.10), coronary artery disease (HR 1.82; 95% CI 1.34–2.48), hypertension (HR 1.24; 95% CI 1.02–1.50), and weekly trastuzumab administration (HR 1.33; 95% CI 1.05–1.68) [58].

Both studies indicated that the rates of trastuzumab-related cardiac events observed in this aged population were higher than those reported in clinical trials. In line with these data, in a study among 68,536 patients with incident breast cancer diagnosed from 1998 to 2007 and who were followed through 2009, a 10% incidence of CMP/CHF (HR = 2.08, 95% CI 1.77–2.44, *p* ≤ 0.001) was observed in cohorts who were 66 years and older and had no prior diagnosis of CMP or CHF [69]. This twofold risk further emphasizes the need to prevent and manage cardiac risks in the elderly who are considered for trastuzumab, as they are likely more vulnerable to its toxicities. Notably, the higher incidence of cardiac events seen in patients treated with adjuvant trastuzumab in “real practice” over clinical trials was evident not only in the elderly but also in the general population [70,71,72,73].

Cardiac dysfunction is a major concern, more so in older patients where toxicity is further magnified when trastuzumab is used in combination with anthracyclines [74]. Therefore, the use of an anthracycline-free regimen could be a key option in older patients with low to intermediate risk of relapse or in patients with comorbidities posing a higher risk for cardiotoxicity.

### 4.2. Adjuvant Trials on Trastuzumab and Anthracycline-Free Combination

In the Breast Cancer International Research Group (BCIRG) study 006, the docetaxel plus carboplatin plus trastuzumab (TCH) regimen showed similar efficacy to standard anthracycline-taxane-trastuzumab regimens but with lesser cardiac toxicity [47]. However, the study had an upper age limit of 70 years, thereby limiting conclusions in much older patients. More importantly, the high dose of carboplatin (AUC 6) in combination with docetaxel would likely make this regimen too difficult for the majority of older patients.

In an open-label phase II study of predominantly node-negative early breast cancer patients, the non-anthracycline docetaxel and cyclophosphamide combined with trastuzumab achieved a 2-year DFS of 97.8% (CI 96.0–98.8) and overall survival of 99.2% (CI 97.8–99.7) [75]. Cardiac dysfunction was seen in 6% of patients (grade 3 in 0.4% of patients) and was generally reversible. The median age group was 55 years in this study but women as old as 75 years were also included, thus making this regimen a suitable option in the elderly.

The combination of weekly paclitaxel and trastuzumab has also been studied in 406 patients with small (≤3 cm), node-negative Her-2 positive disease. Although this was a single-arm phase II study with no specific focus on the elderly population, the relapse rate was encouragingly low (3-year DFS 98.7%), and the combination was relatively non-toxic (0.5% incidence of symptomatic heart failure) [76]. Weekly paclitaxel plus trastuzumab therefore, is a potential treatment alternative for elderly patients with Stage 1 disease or who are not suitable for standard polychemotherapy.

RESPECT (N-SAS BC07) is a prospective phase 3 multicentre trial aiming to compare the efficacy and safety of trastuzumab monotherapy *vs.* standard trastuzumab in combination with chemotherapy [77]. The results are awaited and may be particularly useful for elderly patients with contraindications to or unable to tolerate chemotherapy.

For now, there are no clinical data available for treatment with trastuzumab alone in patients who are not candidates for chemotherapy; however, the 2013 St. Gallen consensus supports that if chemotherapy cannot be given in certain situations, then it might be reasonable to give trastuzumab without it [78].

## 5. Adjuvant Endocrine Therapy

SIOG and EUSOMA recommend adjuvant endocrine therapy to all patients with hormone sensitive tumors given that the efficacy of endocrine therapy is age-independent [79,80]. Aromatase inhibitors (AIs) have been compared with tamoxifen in several large, randomized, adjuvant trials (direct comparison, switch to AI after 2–3 years of tamoxifen, and AI extension after 5 years of tamoxifen) and a small proportion of elderly patients were included (5%–20%). Two analyses were performed specifically in elderly patients. In the BIG 1–98 trial, letrozole showed age-independent superior efficacy compared with tamoxifen [81]. In the MA.17 trial, the advantage conferred by extended letrozole after five years of tamoxifen was significant only in patients younger than 60 years. However, there was no significant interaction found between age and treatment for DFS or OS making extended adjuvant therapy with letrozole a reasonable option for healthy elderly patients [82]. Tamoxifen and AIs have a different safety profile. A systematic review of randomized controlled trials that compared AI and tamoxifen as primary adjuvant endocrine therapy in postmenopausal women revealed that longer AI use was associated with increased odds of developing cardiovascular disease (Odds Ratio {OR} 1.26, 95% CI 1.10–1.43, *p* < 0.001; number needed to harm {NNH} = 132) and bone fractures (OR 1.47, 95% CI 1.34–1.61, *p* < 0.001; NNH = 46); but decreased odds of venous thrombosis (OR 0.55, 95% CI 0.46–0.64, *p* < 0.001; NNH = 79) and endometrial carcinoma (OR 0.34, 95% CI 0.22–0.53, *p* < 0.001; NNH = 258) [83]. Therefore, special attention to comorbidities and potential drug toxicities are essential when planning the optimal endocrine adjuvant strategy in older patients.

Compliance to treatment is crucial as non-adherence to endocrine adjuvant therapy is associated with decreased survival in breast cancer patients [84]. Schlenk *et al.* have reported that up to 50% older adults are non-adherent to their medications [85]. A systematic review by Puts *et al.* reviewed 22 manuscripts with majority focused on women with breast cancer and their adherence to adjuvant hormonal therapy. The adherence rate varied from 52%–100%, but factors influencing non-adherence across studies were inconsistent [86]. Determinants of adherence include factors personal to the patient (such as individual health beliefs, social support, cognitive function and socioeconomic status), factors related to the disease (including severity of symptoms and any stigma associated with the condition), factors related to treatment (schedule, evidence of benefit, side effects), and factors related to the interaction of patient and health care system (such as the quality of the relationship with healthcare providers and convenience of access to facilities) [87]. Given these several factors, individual patient adherence is difficult to predict. But age and side effects are particularly important with oral anticancer therapy [88] and clinicians are urged to consider factors that may result in poor compliance before prescribing oral agents [89]. A recent review of nine U.S. studies of patients aged 65 and older reported that non-adherence was associated with poorer health knowledge and cognitive function, lesser discussion about their condition with the physician, more side effects, and polypharmacy [90]. An adequate cognitive function is also important. A systematic review of studies that looked specifically at older adults with cognitive impairment found that inability to understand new directions, living alone and difficulty scheduling medications into the daily routine were barriers to adherence [91]. Lastly, frailty and non-adherence to adjuvant hormonal therapy in 1,288 older women (median age of 72 years) with breast cancer has been studied prospectively in the CALGB 369901. The study revealed that frailty leads to non-initiation (OR 1.63, 95% CI 1.11–2.4, *p* = 0.013) of hormonal therapy and that the risk of discontinuation increases with age [92].

For patients with a very low-risk tumor (pT1aN0) or life-threatening comorbidities, omission of endocrine therapy could be an option [41]. In a population-based cohort study conducted in Denmark, omission of systemic adjuvant therapy did not affect survival as compared to the general population for women aged 60–74 years, with small (≤10 mm), node-negative, endocrine-responsive, grade 1 ductal carcinoma or grade 1 or 2 lobular carcinoma [93].

## 6. Patient Preference

Patients should be involved as much as possible in treatment decision. In a systematic review of studies that quantified the minimum worthwhile benefits of adjuvant chemotherapy for early breast cancer, a 1% improvement in the chance of survival or cure was considered sufficient to make chemotherapy worthwhile by 50% of women, and a 5% improvement was considered sufficient by 72% [94]. In another study of women’s preferences for considering adjuvant therapy worthwhile, 52%–55% of the women indicated one day or 0.1% sufficient to make adjuvant chemotherapy worthwhile, regardless of the baseline survival [95]. The median age for the studies was 55 years and 28% were age 61–69. There are no data specifically addressing this issue in the elderly and such results may not always hold true for older cancer patients. It is important to emphasize that elderly patient’s preference cannot be accurately predicted by relatives or caregivers due to high discordance between real and perceived needs of the patients [96]. For many fit patients, offering adjuvant treatment when indicated is straightforward. However, in certain situations where the risk and benefits of chemotherapy are equivocal, a good understanding of the clinical evidence while addressing the patient preference are vital to achieve informed decision-making. Although many older patients often rely on the recommendation of their clinicians, it is important to recognize that some may wish to take an active role in decision-making [97,98] and that even if poorly reported in literature, some may choose QoL over quantity [99].

## 7. Conclusions

Fit older breast cancer patients gain as much benefit from adjuvant therapies as younger patients. However, the therapeutic index is generally lower in older than in younger patients because of increased susceptibility to treatment related side effects (especially chemotherapy) associated with aging. Achieving an accurate balance between toxicity and absolute benefit from treatment is necessary. Chemotherapy represents the only treatment option for triple-negative breast cancer patients. For this reason, anthracycline- and taxane-based regimens are recommended for fit elderly patients with high risk N+, triple-negative disease. Less toxic standard regimens (*i.e.*, TC) are recommended for fit patients with N0, triple-negative or HER2+ disease or ER+ disease when chemotherapy is considered. A personalized approach (*i.e.*, single agent chemotherapy for ER-negative and/or N+, HER2+ tumors) is warranted for those who are at high risk for relapse but unfit for a standard regimen. Trastuzumab alone could be an option for patients with high risk HER2+ disease who are unfit for chemotherapy. Endocrine therapy is generally recommended in patients with ER+ disease with a possible omission in patients with low tumor burden (*i.e.*, pT1aN0 disease or life-threatening conditions). A geriatric assessment is a valuable tool in the treatment decision-making process to help define the patients’ health status and to identify patients at high risk of toxicity.

## Abbreviations

AC: Doxorubicin and Cyclophosphamide
ADL: Activities of daily living
AI: Aromatase inhibitors
AML: Acute Myeloid Leukemia
A-T: Anthracycline-Taxanes
AUC: Area under curve
BCIRG: Breast Cancer International Research Group
BCSS: Breast Cancer Specific Survival
BP: Blood Pressure
CALGB: Cancer and Leukemia Group B
CARG: Cancer and Aging Research Group
CASA: Chemotherapy Adjuvant Study for women with advanced Age
CGA: Comprehensive Geriatric Assessment
CHF: Congestive Heart Failure
CI: Confidence Interval
CM: Cyclophosphamide and Methotrexate
CMF: Cyclophosphamide, Methotrexate and Fluorouracil
CMP: Cardiomyopathy
CRASH: Chemotherapy Risk Assessment Scale for High-age patients
CT: Chemotherapy
DFS: Disease Free Survival
EC: Epirubicin and Cyclophosphamide
ECOG: European Cooperative Oncology Group
ELDA: Elderly Docetaxel Adjuvant
EORTC: European Organisation for Research and Treatment of Cancer
Epi: Epirubicin
ER: Estrogen Receptor
EUSOMA: European Society of Breast Cancer Specialists
FAC: Cyclophosphamide, Doxorubicin and Fluorouracil
FASG: French Adjuvant Study Group
FN: Febrile neutropenia
G-CSF: Granulocyte Colony Stimulating Factor
GI: Gastro-intestinal
GU: Genito-urinary
H: Trastuzumab
HERA: Herceptin Adjuvant
HR: Hazard Ratio
HT: Endocrine therapy
IADL: Instrumental Activities of Daily Living
IBCSG: International Breast Cancer Study Group
ICE: Ibandronate with or without Capecitabine in Elderly Patients with Early Breast Cancer
LDH: Lactate dehydrogenase
LVEF: Left ventricular ejection fraction
MDS: Myelodysplastic Syndrome
MMS: Mini-mental status
MNA: Mini Nutritional Assessment
MOS: Medical Outcomes Study
NCCN: National Comprehensive Cancer Network
NNH: Number Needed to Harm
N0: Node negative
N+: Node positive
NSABP: National Surgical Adjuvant Breast and Bowel Project
OR: Odds Ratio
ORR: Overall Response Rate
OS: Overall Survival
QoL: Quality of Life
PLD: Pegylated Liposomal Doxorubicin
PR: Progresterone Receptor
PS: Performance Status
PX: nab-Paclitaxel and Capecitabine
RESPECT: Adjuvant Therapy in Her2 Positive Elderly Breast Cancer Patients
SEER: Surveillance Epidemiology and End Results
SIOG: International Society of Geriatric Oncology
Tam: Tamoxifen
TC: Docetaxel and Cyclophosphamide
TCH: Docetaxel, Carboplatin and Trastuzumab
TN: Triple negative
*vs.*: Versus
+: Positive
